# 初露端倪——演绎ALK阳性非小细胞肺癌的中国故事

**DOI:** 10.3779/j.issn.1009-3419.2015.02.01

**Published:** 2015-02-20

**Authors:** 晓晴 刘

**Affiliations:** 100071 北京，中国人民解放军第三O七医院肺部肿瘤科 Department of Lung Cancer, 307 Hospital of PLA, Affiliated to Academy of Military Medical Sciences, Beijing 100071, China

记得1999年第一次去美国亚特兰大参加美国临床肿瘤学会（American Society of Clinical Oncology, ASCO）年会，当时将“分子靶向治疗”这个还觉陌生的名词仅仅与细胞和动物联系在了一起，感觉“服一片药代替化疗能够治疗肿瘤”就像“天方夜谭”或“美丽的童话”般遥远。而现实是时隔三年的2002年，第一个肺癌分子靶向治疗药物吉非替尼进入临床实践，此后更多分子靶向药物如雨后春笋般涌现。而其中表皮生长因子受体（epidermal growth factor receptor, *EGFR*）突变与EGFR酪氨酸激酶抑制剂（tyrosine kinase inhibitors, TKIs）疗效关系的精彩故事真正掀开了个体化分子靶向治疗的序幕。基于新的理念、新的思维设计的药物及全新的临床试验思路，从2007年肺癌患者ALK靶点发现至2011年针对间变性淋巴瘤激酶融合基因（anaplastic lymphoma kinase, ALK）的抑制剂克唑替尼获得美国食品药品监督管理局（Food and Drug Administration, FDA）许可上市，仅仅用4年的时间，便走完了EGF→EGFR→EGFR-TKI近乎40年的历程，转化性研究速度之快令人惊讶。

随着克唑替尼系列研究的进行，目前晚期非小细胞肺癌（non-small cell lung cancer, NSCLC）个体化靶向治疗正向着纵深不断推进。而PROFILE 1014已于2014年12月4日在新英格兰医学杂志的发表，则标志着ALK阳性NSCLC的全球故事渐入佳境。从开始的尤抱琵琶半遮面，至如今的如火如荼，更多临床研究的拓展使得我们对ALK阳性NSCLC这一类肺癌分子亚型的了解也越发深入透彻。

作为肺癌发病人数的第一大国，中国ALK阳性NSCLC的诊断率已经走到了全球的前列。越来越多的ALK阳性NSCLC患者被确诊，我们对该类疾病的临床特征与治疗方法的理解也正日渐深入。经过近2年的临床经验的积累，国内外重要期刊中，越来越多的中国研究见诸报道。应该说，围绕着如下两条线索，ALK阳性NSCLC的中国故事已经初露端倪！

## 1 引领潮流——更符合中国国情的诊断策略

众所周知，分离探针荧光原位杂交（fluorescence *in situ* hybridization, FISH）一直是ALK阳性NSCLC的经典诊断方法。但是，由于价格较高与技术操作的问题，FISH方法可能不太适合大规模用于ALK诊断。因此，在克唑替尼上市之初，考虑到中国国情，中国专家共识便将Ventana免疫组织化学（immunohistochemistry, IHC）与RT-PCR共同列为了ALK阳性NSCLC的诊断方法，并规范了其操作流程。与之相印证，中国食品药品监督管理局（Chinses Food and Drug Administration, CFDA）也从官方层面批准了上述两种方法临床应用的合理性。由于国内外诊断方法的差异，应该说，从初始临床专家是带着半信半疑的有色眼镜来看待这两种方法的临床应用的。

不过，随着临床实践中经验的积累，越来越多的临床真实案例告诉我们，Ventana IHC与RT-PCR也可以做为ALK阳性NSCLC值得信赖的诊断方法。特别是Ventana IHC，操作简单、价格便宜，越来越为广大临床医生与病理科医师所接受。本专题中，来自郑州大学附属第一医院与南京军区福州总医院的研究，便比对了Ventana IHC与FISH的匹配度。其结果再次验证了Ventana IHC、FISH、RT-PCR均可以成为中国ALK阳性NSCLC患者的标准诊断方法。笔者也欣喜地发现，在诺华公司与罗氏公司设计的二代ALK抑制剂的全球Ⅲ期临床研究中，Ventana IHC已经成为了ALK阳性NSCLC的标准诊断方法，此改变标志着中国专家先知先觉已然走在了欧美同行的前列。

## 2 克唑替尼治疗数据的积累——中国故事的珍贵“素材”

ALK阳性NSCLC是一类由分子基因驱动的恶性肿瘤，ALK抑制剂治疗可获得非常理想的疗效。与EGFR的故事不同，从PROFILE 1001到PROFILE 1029，中国专家与患者便参与了克唑替尼所有重要的Ⅰ期-Ⅲ期临床研究，贡献即亦积累了丰富的治疗经验和体会。本专题中，有关于治疗的两篇个案报道，其一是*EML4-ALK*融合基因阳性的肺腺癌合并淋巴瘤应用克唑替尼治疗成功的病例。另一个案报道了因骨髓转移无法进行常规化疗的ALK阳性晚期NSCLC接受克唑替尼治疗取得了显著疗效，这些珍贵的经验将扩展我们的治疗视野，启发我们在临床实践中勇于探索，以期为更多患者解决治疗难题。

类同于EGFR-TKI，ALK阳性NSCLC同样面临克唑替尼的耐药，关于耐药机理和耐药后治疗策略的综述为我们全面和详尽地描述了近年来耐药机制的研究结果，既合理解释了失败现象，同时提出了耐药后的治疗对策。在临床实践中如果我们能很好地理解克唑替尼的耐药机制，并身体力行地对每位失败或耐药患者原因知晓，然后针对性地给予不同原因耐药后的治疗策略，料想这类晚期NSCLC患者的生存期将得到大大延长。

“依葫芦画瓢”，基于一代EGFR-TKI耐药后的治疗策略，一代ALK抑制剂、二代ALK抑制剂耐药之后，循着这条思路发展下去，*EGFR*突变或者ALK阳性NSCLC，甚至只要有驱动基因存在的晚期NSCLC变成一个慢性病将不会是一个遥远的梦想。

中国患者基数庞大，随着ALK阳性NSCLC诊断率的逐渐提高，ALK阳性晚期NSCLC临床治疗数据的不断积累，我们对于此类疾病的理解正在不断地赶超欧美同行。希望在不远的将来，本专刊带给大家的启示，能够转换成在全球演绎一个属于中国ALK阳性患者长期生存的优美故事，更期冀这些珍贵的中国“素材”在NSCLC个体化治疗的国际舞台上铿锵起唱，留下其浓墨重彩的一笔。

